# HbA1c underperforms in identifying abnormal glucose tolerance in the presence of G6PD deficiency: Insight from the Africans in America study

**DOI:** 10.1371/journal.pone.0334634

**Published:** 2026-01-23

**Authors:** Amy R. Bentley, Kauthrah Ntabadde, Claudine B. Kabeza, Kenneth Ekoru, Christopher W. DuBose, David B. Sacks, Adebowale A. Adeyemo, Charles N. Rotimi, Anne E. Sumner

**Affiliations:** 1 Center for Research on Genomics and Global Health, National Human Genome Research Institute, National Institutes of Health, Bethesda, Maryland, United States of America; 2 Section on Diabetes, Nutrition and Health, Diabetes, Endocrinology, and Obesity Branch, National Institute of Diabetes, Digestive and Kidney Diseases, National Institutes of Health, Bethesda, Maryland, United States of America; 3 Omnigen Biodata Limited, Cambridge, United Kingdom; 4 Clinical Center, National Institutes of Health, Bethesda, Maryland, United States of America; 5 National Institute of Minority Health and Health Disparities, National Institutes of Health, Bethesda, Maryland, United States of America; 6 Hypertension in Africa Research Team, North-West University, Potchefstroom, North-West, South Africa; Shoklo Malaria Research Unit, THAILAND

## Abstract

G6PD deficiency (G6PD-D) variants are associated with lower hemoglobin A1c (HbA1c) concentrations, raising concerns about the diagnostic efficacy of HbA1c for abnormal glucose tolerance (Abnl-GT) in Africans, in whom risk of G6PD-D and Abnl-GT is high. G6PD-D is assessed using genotyping or an enzymatic assay, but because G6PD-D is X-linked, the enzymatic assay is necessary for determining status for women heterozygous for deficiency variants. We assessed: 1) ability of HbA1c to detect Abnl-GT by G6PD-D; 2) concordance of genotyping and enzymatic assay for G6PD-D in sub-Saharan Africans living in the US. 534 participants of the Africans in America study were included, with HbA1c ranging from 3.1–11.3%. Abnl-GT determined by HbA1c (≥5.7%) was compared to the diagnostic standard, the oral glucose tolerance test (fasting glucose≥100 mg/dL and/or 2h glucose≥140 mg/dL). G6PD-D status was determined by genotype (n = 263), enzymatic assay (n = 83), or both (n = 188). G6PD-D could not be determined for 13 women heterozygotes with only genotype data. In the remaining participants, HbA1c was 0.9% lower among those with G6PD-D (4.6 ± 0.5; range 3.1–5.6) compared to those with normal G6PD activity (5.5 ± 0.6; range 4.2–11.3; *P* < 0.001). Glucose concentrations did not differ between groups. HbA1c sensitivity and specificity for Abnl-GT were 0% (0/17) and 100% (37/37) among those with G6PD-D, and 50% (98/195) and 80% (217/272) among those with normal activity. After excluding women heterozygotes, concordance for G6PD-D detection by genotype and the enzymatic assay was 100%. G6PD-D was associated with ~0.9% lower HbA1c in this study, leading to a failure of HbA1c to identify Abnl-GT in these participants. Such a dramatic difference in a screening tool could have consequences in practice, including late diagnosis, undertreatment, and increased complications among those with G6PD-D. Additionally, the results for the enzymatic assay were perfectly concordant with the genotype results for G6PD-D. However, as genotype alone cannot predict G6PD-D in heterozygous women, the enzymatic assay was more informative.

## Introduction

Early diagnosis of type 2 diabetes (T2D) or its antecedent, prediabetes, enhances the opportunity to prevent complications and promote remission. However, the identification of methods to achieve early diagnosis has proven to be challenging. For public health policy as well as optimizing individual care, it is essential to know the strengths and weaknesses of currently available tests.

The oral glucose tolerance test (OGTT) has been used for decades as the glycemia-based diagnostic standard for the detection of abnormal glucose tolerance (Abnl-GT), a summary term for T2D and prediabetes. However, as the OGTT requires blood draw at fasting and two hours, it is labor-intensive and logistically challenging. As an alternative, attention has turned to hemoglobin A1c (HbA1c), a form of glycated hemoglobin, and a non-fasting measure of glycemia over the previous two to three months. Since 1979, HbA1c has been used in individuals with diabetes to monitor glycemic control. Since 2011, with the resolution of issues related to the HbA1c assay, HbA1c has been used for diagnosis of T2D (≥6.5%) and prediabetes (≥5.7%). However, the effectiveness of HbA1c as a diagnostic test has been challenged. In sub-Saharan African countries, the diagnostic sensitivity of HbA1c has been found to be only about 50% [1]. The explanation for this phenomenon has been elusive.

Large-scale epidemiological analyses, including the Meta-Analyses of Glucose and Insulin related traits Consortium (MAGIC), have identified African ancestry-specific variants that are associated with large reductions in HbA1c (0.7–1.0%) [2–4]. These variants are a part of the A- haplotype in the glucose-6-phosphate dehydrogenase gene (*G6PD*) that causes G6PD deficiency (G6PD-D). *G6PD* A- is responsible for the major form of G6PD-D in Africa and is common in malaria-endemic regions of the continent [5]. It is widely accepted that the prevalence of this haplotype is due to natural selection for protection against malaria [6].

Importantly, the lower values of HbA1c with *G6PD* A- do not accurately reflect glycemia. G6PD is an enzyme in the pentose phosphate pathway and is crucial for protecting red blood cells from oxidative damage [7]. G6PD-D leads to hemolysis and shorter red blood cell lifespan. This more rapid turnover of red blood cells causes lower HbA1c levels discordant with the degree of glycemia, as has been observed with the Sickle Cell mutation [8,9]. Thus, *G6PD* A- could contribute to population differences in the efficacy of HbA1c as a diagnostic test and contribute to health disparities. The MAGIC investigators postulate that 2% of African Americans with T2D are undiagnosed as a result of HbA1c screening in the United States missing those with G6PD-D [4]. Relatedly, in a meta-analysis of nearly 200,000 participants with T2D, *G6PD* A- variant rs1050828-T was significantly associated with diabetic retinopathy and other T2D complications [10]. This effect was fully mediated by glycemia, supporting the idea that reliance on HbA1c for diagnosis and treatment can lead to late diagnosis, undertreatment, and more T2D complications for those with G6PD-D. Unfortunately, OGTT measurements were not available in these previous large studies to evaluate the effect of G6PD-D diagnostic capability of HbA1c for Abnl-GT compared to the diagnostic standard, which was identified as a need [4,10].

G6PD-D can be determined using either a test of G6PD enzyme activity or by genotyping for *G6PD* variants known to cause deficiency. Each of these methods has limitations in different contexts. In malaria-endemic regions, individuals with deficiency may appear to have normal G6PD activity using an enzymatic test, potentially due to more rapid red blood cell turnover caused by infection [11]. Similarly, failure to identify low G6PD activity using the enzymatic test has been observed among patients whose treatments affected hematological parameters [12]. However, as *G6PD* is on the X chromosome, women experience random inactivation of one copy of *G6PD*. Thus, in women who are heterozygous for *G6PD* A-, genotyping cannot predict whether the copy causing deficiency will be active and G6PD-D status cannot be determined. Additionally, while presence of the *G6PD* A- haplotype is largely determined by homozygosity for rs1050828-T and screening for this single variant identifies the vast majority of *G6PD* A- individuals, the haplotype also includes rare variants rs76723693 and rs137852328, which are genotyped less commonly for screening. Thus, prevalent screening methods by genotype may miss individuals that the enzymatic assay would identify.

To contribute primary data in Africans on the influence of G6PD-D on the diagnostic value of HbA1c, we turned to the Africans in America Study. The enrollees are sub-Saharan Africans currently living in the metropolitan Washington, DC area. Study procedures include OGTT, HbA1c measurement, and the assessment of G6PD-D by both genotyping and the enzymatic assay for G6PD activity. In this study our goals were to determine: 1) the ability of HbA1c to detect Abnl-GT by G6PD-D status, and 2) test the concordance between the enzymatic assay and genotyping for *G6PD* risk variants for determining G6PD-D.

## Materials and methods

The Africans in America study was designed to identify effective screening tests for diabetes and cardiovascular disease in sub-Saharan Africans living in the United States [13–16]. The protocol was approved by the Institutional Review Board of the National Institutes of Health (NIH; Clinical Trials.gov Identifier: NCT00001853). Participants for this protocol were recruited from 6/13/2011 to 12/12/2024. Participants gave written consent.

Recruitment was achieved with flyers and posters and presentations at community events, announcements in the NIH Clinical Trials website, advertisements in a local newspaper and on social media. Interested persons called the study line and participated in a telephone interview to determine eligibility. Potential enrollees had to be currently living in the Washington, DC metropolitan area, be between 18 and 70 years of age, report no known history of diabetes, state that they were born in a sub-Saharan African country, and that both their parents self-identified as “Black” and were also born in sub-Saharan African country.

**Fig 1:**
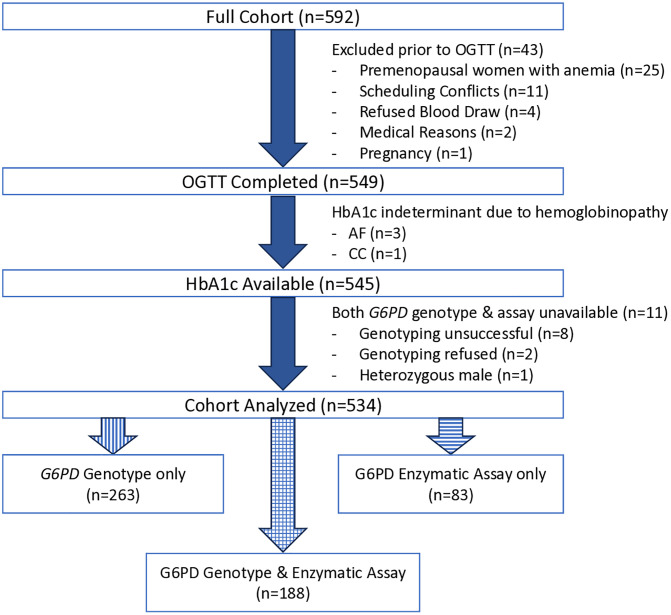
Flow chart. Abbreviations: OGTT (Oral Glucose Tolerance Test), HbA1c (hemoglobin A1c), G6PD (glucose-6-phosphate dehydrogenase).

After eligibility was confirmed by the telephone screening interview, 592 individuals came to the NIH Clinical Center for a history and physical, routine blood tests, including a complete blood count and iron studies, and an electrocardiogram. Of the 592 participants, 93% (549/592) were invited to return within 2 weeks or less for an OGTT (Fig 1). The reasons for not returning for an OGTT included: anemia (n = 25), scheduling conflicts (n = 11), blood draw refusal (n = 4), pregnancy (n = 1), and medical reasons (n = 2), such as newly diagnosed hypothyroidism.

OGTT were performed in 549 enrollees (Trutol 75, Custom Laboratories, Thermo Fisher Scientific, Middletown, VA). Abnl-GT was defined by fasting glucose ≥ 100 mg/dL and/or 2h glucose ≥ 140 mg/dL and T2D was defined by fasting glucose ≥ 126 mg/dL and/or 2h glucose ≥ 200 mg/dL. In addition, plasma was obtained for determination of B12 and folic acid levels. Whole blood was obtained for hemoglobin electrophoresis and HbA1c was measured by an NGSP-certified method (HPLC, BioRad Laboratories, Hercules, CA). HbA1c ≥ 5.7% was the threshold for Abnl-GT. After the OGTT, 4 individuals were excluded because hemoglobinopathies made HbA1c values indeterminate. Three of the four persons had increased hemoglobin F and one person had hemoglobin CC.

Thus, 545 people were available for analysis of their G6PD status by either genotype, enzymatic assay or both. However, G6PD status could not be determined for 11 persons, as they did not have the enzymatic assay performed and their *G6PD* genotype could not be established, and these were excluded. Genotyping was unsuccessful in 8 of the 11 individuals. Of the 3 remaining persons, 2 refused genotyping and one was a heterozygous male (suggesting an error, as males have a single copy of the X chromosome). Therefore, G6PD-D status could be evaluated in 90% (534/594) of the original cohort. All participants with G6PD-D were unaware of their status prior to this study.

### G6PD-D analyses

G6PD-D status was determined by genotype only in 49% (263/534), by enzymatic assay only in 16% (83/534) and by both methods in 35% (188/534; Fig 1).

The enzymatic assay for G6PD activity used qualitative visual colorimetric determination fluorescence screening in red blood cells (Trinity Biotech Procedure No. 400 Kit, Jamestown, NY, USA).

Genotyping was conducted using either Illumina’s Multi-Ethnic Genotyping Array or the Global Diversity Array. The *G6PD* A- haplotype was defined separately in men and women. Since the *G6PD* locus is on the X chromosome, the haplotype in women is determined by alleles on both X chromosomes, while haplotype in men is determined by genotype at one X chromosome. Men were defined as having *G6PD* A- if they were hemizygous for the C allele at rs1050829 and hemizygous for any of the following: the T allele at rs1050828, the C allele at rs76723693, or the A allele at rs137852328. Women were defined as having the haplotype similarly, except by homozygosity for the rs1050829 risk allele as well as homozygosity for the risk allele at any of the other 3 *G6PD* A- variants. Women were defined as being heterozygous for *G6PD* A- if they were at least carriers of the risk allele at rs1050829 and also at one of the other *G6PD* A- variants, but not homozygous for both. Detailed *G6PD* A- allele counts for all genotyped individuals is given in **S2 Table**.

### Statistical analyses

A *P*-value ≤0.05 was considered statistically significant. Unless stated otherwise, results are presented as mean ± SD. Comparisons by African region of origin were tested using one-way ANOVA. Comparisons by gender or G6PD status were by unpaired t-test, Mann-Whitney or chi-square test, as appropriate. Research Electronic Data Capture (REDCap) was used for data management (Harris PA 2009). STATA v18 (College Station, Texas) was used for data analyses. R 4.2.2 (https://cran.r-project.org/) was used for visualization of data (ggplot2, rnaturalearth).

## Results

### Cohort demographics

**Fig 2:**
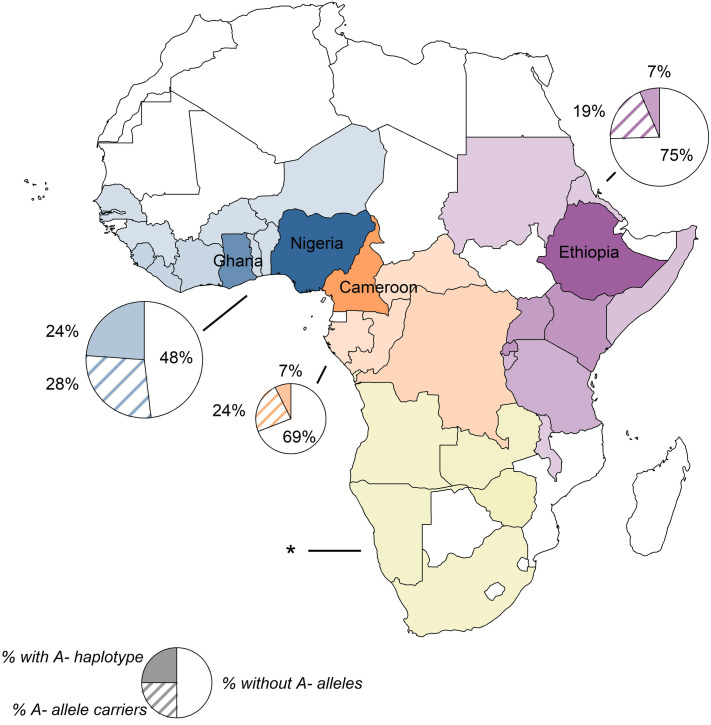
Birth countries of participants and distribution of G6PD A- haplotype. Birth countries of the 534 participants colored based on region (West n = 249): blue; Central (n = 76): orange; East (n = 195): purple; South (n = 14): yellow) with shading proportional to number of participants included from that country (darker shades = more participants). Pie charts show the distribution of G6PD A- alleles in participants from each region for all those with genotype data (n = 451). The size of the pie chart is proportional to the number of participants from that region. *Due to low number of participants from the South of Africa with genotype data (n = 12), no pie chart is shown.

The African region of origin of the participants were: West 47%, East 36.5%, Central 14%, and South 3% (**Table 1**; Fig 2). The mean age of enrollees was 40 ± 11y, range 20 to 70y. Mean BMI was 28.0 ± 4.6, range (19.3 to 46.2 kg/m^2^). Most of the immigrants were male (61%), arrived in the United States since the year 2000 (68%) and were from West (47%) or East Africa (36.5%) (**Table 1**). Sickle cell trait occurred in 15% of participants and was more common in individuals from West and Central Africa than East Africa (*P* < 0.001). Hemoglobin C trait occurred exclusively in participants from West Africa (*P* = 0.001).

**Table 1:** Participant demographics.

Characteristic	Total 100% (n = 534)	West 47% (n = 249)	East & South^a^ 39% (n = 209)	Central 14% (n = 76)	*P*-value^b^
**Age (y)**	40 ± 11	41 ± 11	39 ± 10	41 ± 12	0.127
**BMI (kg/m**^**2**^)	28.0 ± 4.6	28.6 ± 4.6	27.0 ± 4.6	28.8 ± 5.3	<0.001a***c**
**Male (%)**	61%	62%	55%	70%	0.059
**Years of US Residence**	12 ± 10	14 ± 11	11 ± 9	13 ± 10	<0.001a**
**Arrived in ≥ 2000**	69%	65%	73%	69%	0.179
**Sickle Cell Trait (%)**	15%	19%	8%	22%	<0.001
**HbC trait (%)**	2%	5%	0%	0%	0.001
**G6PD-D (%)** ^**c**^	10%	16%	5%	8%	<0.001

^a^Individuals from Southern African countries (n = 14) were combined with the East Africa group.

^b^One Way ANOVA or chi-square as appropriate, a: comparison of West vs. East, b: West vs. Central, c: East vs. Central. *P ≤ 0.05, **P ≤ 0.01, ***P ≤ 0.001.

^c^Thirteen women were excluded because the enzymatic assay was not available, and G6PD-D could not be determined by genotype due to heterozygosity for *G6PD* A-.

### Prevalence of G6PD deficiency in analytic sample

There were 534 individuals whose G6PD-D status was evaluated by either genotyping, the enzymatic assay or both. In the group who had genotyping only (n = 263), 13 women were found to be heterozygous for *G6PD* A-, meaning that their G6PD-D status could not be established by genotyping alone. As these women did not have the enzymatic assay, their functional G6PD-D status was indeterminate.

In the remaining 521 participants, based on either genotyping or enzymatic assay results, G6PD-D occurred in 10% (54/521). G6PD-D prevalence in men and women was 14% (45/324) vs. 5% (9/197), *P* < 0.001, respectively, with the sex-biased distribution expected of an X-linked trait. By African region of origin, the frequency of G6PD-D was West (16%), East (5%), and Central (8%) (*P* < 0.001). Among those from West Africa, over half either had the *G6PD* A- haplotype or were carriers of *G6PD* A- variants (Fig 2), and 70.4% of study participants with *G6PD* A- were from West Africa (S1 Table), consistent with the known distribution of the haplotype. Ninety-five % of those with the *G6PD* A- haplotype were homo-/hemizygous for rs1050828-T (in addition to rs1050829-C), however 2 participants with the haplotype were homo-/hemizygous for rs76723693-C; additionally, one woman was heterozygous for rs137852328-A (see Methods for haplotype definition; S2 Table).

### Abnormal glucose tolerance status

Abnl-GT, as determined by OGTT, occurred in 41% (212/521) of participants. Of those, 88.8% (191/215) were classified as Abnl-GT based on 2-hour plasma glucose ≥ 140 mg/dl and 36.3% (78/215) were classified based on fasting plasma glucose ≥ 100 mg/dl. The prevalence of Abnl-GT in participants with and without G6PD-D was 32% (17/54) vs. 42% (195/467), respectively, *P* = 0.146. Of the 17 persons, with both G6PD-D and Abnl-GT, T2D occurred in 23% (4/17) and prediabetes in 77% (13/17). Of the 195 individuals with normal G6PD-D activity and Abnl-GT, T2D occurred in occurred in 19% (37/195) and prediabetes in 81% (158/195).

### HbA1c levels

**Fig 3:**
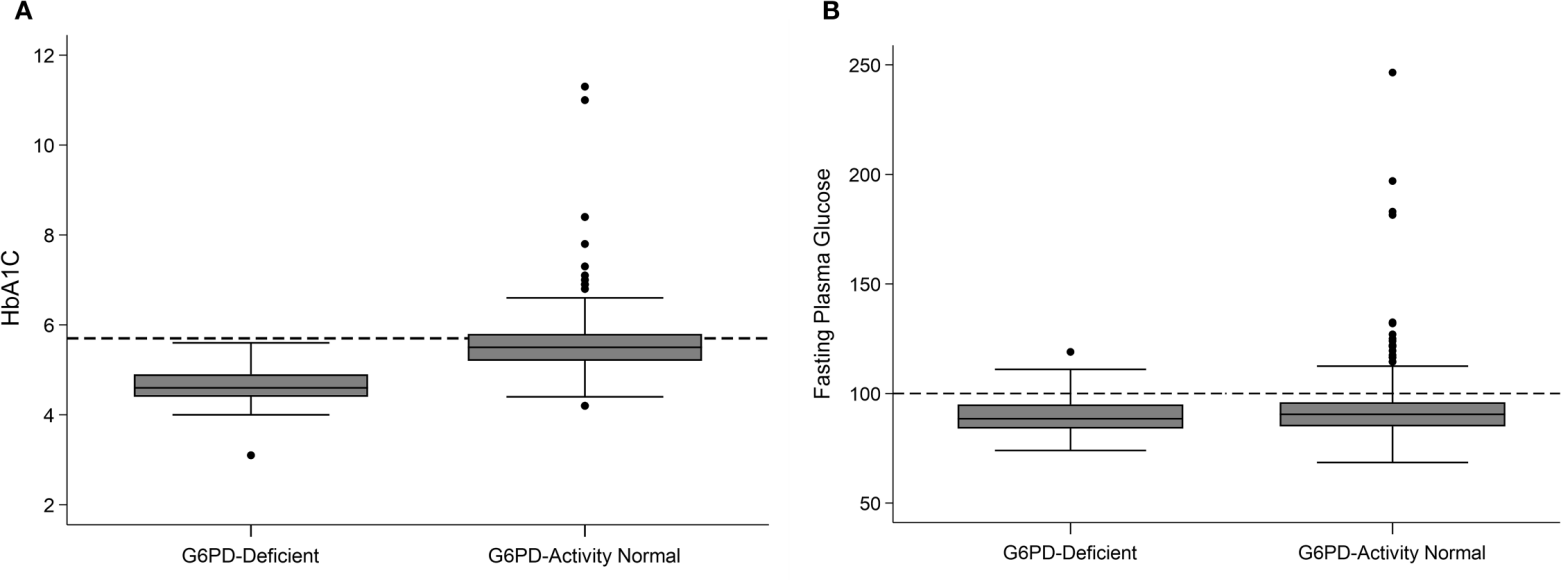
HbA1c and fasting glucose by G6PD-D. Box and whiskers plots of: **A)** HbA_1C_ by G6PD-D. Dotted line at HbA1C=5.7%. *P* < 0.001; **B)** Fasting Plasma Glucose by G6PD-D. Dotted line at Glucose = 100 mg/dL. *P* = 0.481.

HbA1c levels in those with G6PD-D were 4.6 ± 0.5%, (range 3.1 to 5.6), 0.9% lower than among those with normal G6PD-D activity (5.5 ± 0.6%; range 4.2 to 11.3; *P* < 0.001; Fig 3A). Of note, no participant with G6PD-D had HbA1c ≥ 5.7%, thus, none were identified as having Abnl-GT based on HbA1c. Importantly, among the participants with Abnl-GT who were missed were 4 individuals with T2D, as determined by OGTT. While HbA1c was lower in the group with G6PD-D, there were no statistical differences between groups in either fasting glucose (*P* = 0.481; Fig 3B) or 2h glucose, (*P* = 0.220). With G6PD-D, HbA1c sensitivity for diagnosis of Abnl-GT was 0% (0/17) and specificity was 100% (37/37). With normal G6PD-D activity, sensitivity was 50% (98/195) and specificity was 80% (217/272).

### Measures of hemolysis

Measures of hemolysis, specifically reticulocyte percent and absolute reticulocyte count were available in 475 consecutively enrolled individuals. Reticulocyte percent was higher in the G6PD-D group than the group with normal G6PD activity (2.92 ± 0.61 vs. 1.45 ± 0.42, *P* < 0.001). Similarly, absolute reticulocyte count was higher in the group with G6PD deficiency compared to the group with normal G6PD activity (94.1 ± 27.6 vs. 70.4 ± 20.6, P < 0.001).

### Concordance of G6PD identification by genotyping and enzymatic assay

There were 188 participants with both enzymatically measured G6PD and *G6PD* genotyping available (Fig 1). Of these, 15 were women who were heterozygous for the *G6PD* A- haplotype, so their G6PD-D status could not be determined based on genotyping, and they were excluded from the analysis. In the remaining 173 participants, there was perfect concordance between haplotype prediction and enzymatic assay: all those with the *G6PD* A- haplotype had deficiency based on the enzymatic assay and all those who did not have the haplotype had normal G6PD activity. Thus, genotyping and the enzymatic assay performed equally well to screen for G6PD-D among men.

## Discussion

In the Africans in America cohort, we evaluated the ability of HbA1c to detect Abnl-GT in the presence and absence of G6PD deficiency, using OGTT as our diagnostic standard. We had three main findings: (1) HbA1c failed to diagnose Abnl-GT in individuals with G6PD-D, including individuals who had T2D; (2) G6PD-D lowered HbA_1C_ levels by ~0.9% on average and (3) for the detection of G6PD-D, the enzymatic assay was more informative than genotyping. These results highlight that the reduction of HbA1c associated with G6PD-D is clinically significant and of great public health importance.

### Sensitivity and specificity of HbA_1C_ with G6PD-deficiency

In the group with G6PD deficiency the mean HbA1c was 4.6 ± 0.5% and the range was 3.1 to 5.6%, meaning the span was just 2.5% (Fig 3A). Therefore, in the presence of G6PD-D, the sensitivity of HbA_1C_ was 0%. This means if HbA1c was the sole test used for Abnl-GT screening, no individual with both Abnl-GT and G6PD-D would have been identified. As a consequence of the 0% sensitivity, specificity was 100%. As no person with G6PD-D and normal glucose tolerance crossed the HbA1C ≥ 5.7% threshold, there were no false positives. While a high specificity from a low rate of false positives is good; the public health cost is high. With high specificity and low sensitivity, undiagnosed individuals with Abnl-GT are not identified and not offered either early intervention or treatment.

Early identification of Abnl-GT is of high priority to reduce the development of T2D complications, and the impact of hyperglycemia on other cardiometabolic traits. The use of HbA1c as a screening tool will lead to failure to identify individuals with Abnl-GT who have G6PD deficiency. In the present study, 10.3% of participants had G6PD-D, meaning that G6PD-D could affect the utility of HbA1c in 1 in 10 such sub-Saharan African immigrants. This would contribute to disparities in Abnl-GT detection as well as in the numerous conditions that delayed identification of hyperglycemia could worsen. The impact of G6PD-D on T2D complications has been observed in a recent study of African Americans in the ACCORD Trial and the Million Veterans Program [10]. Individuals with *G6PD* A- variant rs1050828-T had a higher risk of retinopathy than those without the deficiency at the same HbA1c levels. Importantly, this increased risk was completely mediated by glycemia, with mediation analysis showing no evidence for a direct effect of rs1050828-T on diabetic retinopathy (direct effect *P* = 0.95), indicating that this association results from a failure to detect hyperglycemia in individuals with G6PD deficiency because of reduced levels of HbA1c[10]. It is important to note that T2D complications can occur prior to reaching the thresholds for T2D diagnosis [17], emphasizing even further the need for accurate and early identification of those with Abnl-GT.

Researchers have recommended that HbA1c values (or diagnostic thresholds) be adjusted for those with G6PD-D[2, 4, 10]. In our samples, simply adjusting the HbA1c levels in individuals with G6PD-D by the mean 0.9% difference between those with G6PD-D and those with normal G6PD activity led to a considerable improvement in diagnostic capacity of HbA1c for Abnl-GT: a sensitivity of 70.6% and specificity of 81%, compared to 0% and 100%, respectively, for unadjusted HbA1c levels. Such a dramatic improvement with even this simple adjustment suggests that this is a promising avenue for future research.

### Sensitivity and specificity of HbA1c in presence of normal G6PD activity

In the group with normal G6PD activity the mean HbA1c was 5.5 ± 0.6% and the range was 4.2 to 11.3%, meaning the span was 7.1%. For participants in the Africans in America cohort with normal G6PD activity, the sensitivity of HbA1c was 50% and the specificity was 80%. While a sensitivity of 50% for HbA1c in the group with normal G6PD activity is suboptimal, it is substantially better than the 0% sensitivity for HbA1c that was observed among those with G6PD-D.

The sensitivity of HbA1c for diagnosing Abnl-GT in the normal G6PD activity group was similar to studies conducted in Africa. In a meta-analysis of 6,183 participants from 7 African countries the pooled sensitivity for HbA1c for the diagnosis of T2D (with OGTT as the diagnostic standard) was 57% and the specificity was 92% [1]. Therefore, Chivese et al. state that using HbA1c means missing the diagnosis of T2D in almost half of affected individuals. Therefore, even for studies conducted in Africa with much larger cohorts than in our African immigrant study, the sensitivity of HbA1c to the diagnosis of DM is not optimal but it is markedly worse in the G6PD-D group than the group with normal G6PD activity.

### Genotyping for *G6PD* A-

Although the *G6PD* A- haplotype includes 4 variants (rs1050829-C, rs1050828-T, rs76723693-C, and rs137852328-A), when genotyping for G6PD-D in Africa, it is common to evaluate only a subset of these: either only rs1050828 [18] or rs1050828 and rs1050829 [19–21]. Although homo-/hemizygosity for rs1050829-C is necessary for the *G6PD* A- haplotype, the other *G6PD* A- variants are rarely found in the absence of rs1050829-C, and rs1050828-T is thought to have occurred on the background of rs1050829-C [22], thus presence or rs1050829-C can be presumed by presence of one of the other variants. Of these other risk variants, rs1050828-T is by far the most common in Africa, with a minor allele frequency of 12% among African/African Americans in the gnomAD database compared to <1% for rs76723693-C and rs137852328-A (https://gnomad.broadinstitute.org/; though the frequency of rs76723693-C is 6% among Gambians [1000 Genomes GWD] [23]). Thus, genotyping only rs1050828 is expedient for screening for *G6PD* A-, as presence of rs1050829-C can be assumed with rs1050828-T[22] (as seen in our data), and the other two variants account for far less of the deficiency. In our dataset, however, this strategy would have failed to identify two individuals with *G6PD* A- who were homo-/hemizygous for rs1050829-T and rs76723693-C as well as a woman who was heterozygous for rs1050829-T and rs137852328-A. Thus, while this strategy is expedient, it may miss some at-risk individuals.

### Concordance between enzymatic assay and genotyping

The observed perfect concordance between G6PD-D predicted by haplotype and determined by enzymatic assay, after exclusion of women heterozygous for *G6PD* A-, is an important contribution of this work. Much of previous concordance literature has been done in the context of malaria, which has been proposed to mask G6PD-D in clinical measurements and to lead to reduced concordance with genotype data. For instance, 10% of males and 24% of females with the *G6PD* A- haplotype (i.e., women who were homozygous for *G6PD* A-) had normal G6PD activity in a large-scale clinical trial of malaria patients in 6 African countries [11]. Our observed perfect concordance in a sub-Saharan African population living in a non-malarial environment supports the idea that these previous concordance estimates were affected by the presence of malaria-associated hemolysis. Discrepancies between genotyping and enzymatic assay were also observed in the context of a US patient population, where they were affected by hematological factors that are more common among individuals experiencing intensive medical treatment [12]. In our general population sample in a non-malarial environment, the enzymatic assay accurately identified G6PD-D. Given the perfect concordance in this context, the presence of rare *G6PD* A- genotypes that might be missed by some genotyping strategies, and the inability to predict deficiency status in heterozygous females, the enzymatic assay is more informative and simpler to use to determine G6PD-D.

### Strengths and weaknesses

This investigation had several strengths. It is the first to evaluate the effect of G6PD-D on the diagnostic sensitivity of HbA1c against the OGTT in Africans. In addition, we had the opportunity to compare the performance of the enzymatic assay and genotyping for determining G6PD-D in an area where malaria is not endemic. Third, our study was in African immigrants, so likely to be applicable to Africans living in the Diaspora.

The main weakness of this study is the sample size of the Africans in America cohort (n = 534) with G6PD-D occurring in 10%). With 0% sensitivity for Abnl-GT in our group with G6PD-D, with a larger sample size and more advanced Abnl-GT at diagnosis, we would expect a fraction of participants with G6PD-D and Abnl-GT to have higher HbA1c levels than the 5.6% we observed.

In addition, from two key perspectives the Africans in America cohort was representative of: (1) African immigrants in the United States and (2) HbA1c patterns. First, for the African immigrant community, we believe the cohort is representative because, consistent with United States census data, the majority of the immigrants in the Africans in America study were male, entered the United States after the year 2000 and were mainly from West and East African countries. Second, from the perspective of HbA1c patterns, previous studies found that G6PD-D variants lowered HbA1c levels by 0.7–1.0% [2–4], which matches the 0.9% lowering observed in the Africans in America cohort. In addition, studies in African populations have found that HbA1c sensitivity for the diagnosis of diabetes is ~ 50% [1], just as we found in our participants without G6PD-D.

In conclusion, G6PD-D is associated with clinically meaningful reductions in HbA1c. The use of HbA1c as a diagnostic test for Abnl-GT in the presence of G6PD-D leads to lost opportunity for the early detection of T2D and prediabetes. Therefore, working with an African immigrant population who did not have malaria, we endorse the recommendation of MAGIC investigators that screening for G6PD-D be conducted when HbA1c is going to used diagnostically. Due to challenges with determining G6PD-D by genotyping, including the presence of rare genotypes and the indeterminate G6PD-D status of heterozygous women, we recommend the use of the enzymatic assay and note the availability of hand-held point-of-care quantitative G6PD assays which will facilitate greater screening efficiency.

## Supporting information

S1 Table Distribution of countries of origin among participants with and without *G6PD* A- variants (n = 451). (DOCX)

S2 Table *G6PD* variant allele counts (N = 451). Shown are the number of risk alleles observed for each variant making up the *G6PD* A- haplotype along with the counts of individuals with that distribution.
(DOCX)
