# Supplementary material for: HbA1c underperforms in identifying abnormal glucose tolerance in the presence of G6PD deficiency: Insight from the Africans in America study
Source: PLoS One. 2026 Jan 23;21(1):e0334634. doi: 10.1371/journal.pone.0334634 (PMC12829870; doi:10.1371/journal.pone.0334634)
Supplement: S1 Table — (DOCX) [file pone.0334634.s001.docx]

**S1 Table. Distribution of Countries of Origin among Participants with and without *G6PD* A- Variants (n=451)^1^**

| **Haplotype (n)** | ***G6PD* A- Risk Alleles Carried** | **Region (n; %)^2^** | **Countries (n)** |
| --- | --- | --- | --- |
| *G6PD* A-^3^ (71) | rs1050829-C & rs1050828-T | West (50; 70.4%) | Nigeria (26), Ghana (14), Sierra Leone (3), Ivory Coast (2), Liberia (2), Togo (2), Benin (1) |
|  |  | East (10; 14.1%) | Tanzania (3), Uganda (3), Kenya (2), Rwanda (2) |
|  |  | Central (4; 5.6%) | Democratic Republic of the Congo (2), Cameroon (1), Republic of the Congo (1) |
|  |  | South (4; 5.6%) | Zimbabwe (3), Zambia (1) |
|  | rs1050829-C & rs76723693-C | West (1; 1.4%) | The Gambia (1) |
|  |  | Central (1; 1.4%) | Cameroon (1) |
|  | rs1050829-C & rs137852328-A | West (1; 1.4%) | Nigeria (1) |
| Carriers of a single *G6PD* A- variant^4^ (109) | rs1050829-C | West (61; 56.0%) | Nigeria (27), Ghana (24), Sierra Leone (3), Ivory Coast (2), Benin (1), The Gambia (1), Liberia (1), Senegal (1), Togo (1) |
|  |  | East (29; 26.6%) | Uganda (8), Rwanda (6), Kenya (6), Burundi (3), Ethiopia (3), Tanzania (3) |
|  |  | Central (16; 14.7%) | Cameroon (11), Democratic Republic of the Congo (3), Central African Republic (2) |
|  |  | South (3; 2.8%) | Namibia (1), Zambia (1), Zimbabwe (1) |
| No *G6PD* A- variants (271) |  | East (114; 42.1%) | Ethiopia (41), Rwanda (20), Kenya (16), Burundi (10), Uganda (10), Somalia (7), Tanzania (5), Eritrea (2), Malawi (2), Sudan (1) |
|  |  | West (105; 38.7%) | Nigeria (46), Ghana (28), Liberia (8), Ivory Coast (6), Sierra Leone (5), Togo (3), Burkina Faso (2), The Gambia (2), Senegal (2), Benin (1), Guinea (1), Niger (1) |
|  |  | Central (47; 17.3%) | Cameroon (42), Democratic Republic of the Congo (4), Republic of the Congo (1) |
|  |  | South (5; 1.8%) | Zimbabwe (3), Angola (1), South Africa (1) |

*^1^Table includes all individuals with genotype data; ^2^Counts and percentages within each haplotype group; ^3^Includes G6PD A- Heterozygotes; ^4^There were no carriers of rs1050828, rs76723693, or 137852328 that did not have the G6PD A- haplotype.*
