# Supplementary material for: HbA1c underperforms in identifying abnormal glucose tolerance in the presence of G6PD deficiency: Insight from the Africans in America study
Source: PLoS One. 2026 Jan 23;21(1):e0334634. doi: 10.1371/journal.pone.0334634 (PMC12829870; doi:10.1371/journal.pone.0334634)
Supplement: S2 Table — Shown are the number of risk alleles observed for each variant making up the G6PD A- haplotype along with the counts of individuals with that distribution. (DOCX) [file pone.0334634.s002.docx]

**S2 Table. *G6PD* Variant Allele Counts (N=451)^1^:** Shown are the number of risk alleles observed for each variant making up the *G6PD* A- haplotype along with the counts of individuals with that distribution.

| *G6PD A- Haplotype defined by Hemi-/Homozygosity for the risk allele at:* | | | | | | | | | | |  | |
| --- | --- | --- | --- | --- | --- | --- | --- | --- | --- | --- | --- | --- |
|  | | | *rs1050829* | |  | | *AND any of the following:* | | | |  | |
| **Sex** | **A-** | **rs1050829-C** | |  | | **rs1050828-T** | | **rs76723693-C** | **rs137852328-A** | **Count** | |  |
| Male^2^ | Y | 1 | |  | | 1 | | 0 | 0 | 37 | |  |
|  | Y | 1 | |  | | 0 | | 1 | 0 | 1 | |  |
|  | N | 0 | |  | | 0 | | 0 | 0 | 188 | |  |
|  | N | 1 | |  | | 0 | | 0 | 0 | 57 | |  |
|  |  |  | |  | |  | |  |  |  | |  |
| Female^3^ | Y | 2 | |  | | 2 | | 0 | 0 | 4 | |  |
|  | Y | 2 | |  | | 0 | | 2 | 0 | 1 | |  |
|  | Het | 2 | |  | | 1 | | 0 | 0 | 9 | |  |
|  | Het | 1 | |  | | 1 | | 0 | 0 | 18 | |  |
|  | Het | 1 | |  | | 0 | | 0 | 1 | 1 | |  |
|  | N | 0 | |  | | 0 | | 0 | 0 | 83 | |  |
|  | N | 1 | |  | | 0 | | 0 | 0 | 50 | |  |
|  | N | 2 | |  | | 0 | | 0 | 0 | 2 | |  |

*^1^All individuals with genotype data are included. ^2^As the G6PD locus is on the X chromosome, males can have a maximum of 1 risk allele for each variant. ^3^For females, X-inactivation of one allele means that individuals with 2 or 0 copies of the risk allele at a variant can be expected to have or not have, respectively, that risk allele, while the expressed allele for heterozygotes is uncertain.*
